# Socioeconomic Status Modifies the 20-Year Association Between Metabolic Syndrome and Incident Coronary Artery Disease: A Nationwide Cohort Study of Korean Adults

**DOI:** 10.3390/jcm15176562

**Published:** 2026-08-25

**Authors:** U Chul Ju, Ja Young Kim, Hyun Yi Kook, Yeon Ji Seong, Ho Goon Kim, Eujene Jung

**Affiliations:** 1 Department of Gynecology and Oncology, Chonnam National University Medical School, Gwangju 61748, Republic of Korea; 2 Department of Nursing, Nambu University, Gwangju 62271, Republic of Korea; 3 Department of Emergency Medicine, Chonnam National University Medical School, Gwangju 61748, Republic of Korea; 4 Department of Gastrointestinal Surgery, Chonnam National University Medical School, Gwangju 61748, Republic of Korea

**Keywords:** metabolic syndrome, socioeconomic status, coronary artery disease, health disparities, cohort study, National Health Insurance Service, risk stratification, emergency medicine

## Abstract

**Background/Objectives**: Metabolic syndrome (MetS) and low socioeconomic status (SES) are each established drivers of coronary artery disease (CAD), a leading cause of acute cardiovascular presentations to emergency and acute care services. Whether SES modifies the long-term association between MetS and CAD in a universal health coverage setting is unclear. We examined the independent and joint associations of MetS and income-based SES with 20-year incident CAD. **Methods**: In this nationwide retrospective cohort study using the Korean National Health Insurance Service database, 489,521 adults aged 40–79 years who underwent a national health examination in 2005 and were free of CAD at baseline were followed through 31 December 2024 (up to 20 years). At baseline, the mean age was 54.1 ± 9.2 years, and 252,818 participants (51.6%) were men. MetS was defined by modified harmonized criteria, with body mass index (BMI) ≥ 25 kg/m^2^ substituted for waist circumference. SES was measured by health insurance premium deciles and classified as high (deciles 8–10), middle (4–7), low income (1–3), and Medical Aid (decile 0). The outcome was incident CAD (myocardial infarction or angina pectoris). Multivariable Cox proportional hazards models were adjusted for age, sex, smoking, alcohol consumption, regular exercise, BMI, chronic kidney disease, alternate exposure, and estimated adjusted hazard ratios (aHRs); joint exposure and additive interaction (relative excess risk due to interaction, RERI) were assessed. **Results**: MetS prevalence was 30.6%. During follow-up, 48,428 participants (9.9%) developed CAD. MetS was independently associated with CAD (aHR 1.84; 95% CI 1.78–1.91), as was a graded socioeconomic gradient (Medical Aid vs. high SES aHR 1.62; 1.54–1.70). Incidence rates ranged from 3.13 (high-SES without MetS) to 11.40 (Medical Aid with MetS) per 1000 person-years. In the joint analysis, Medical Aid beneficiaries with MetS had the highest risk (aHR 2.92; 2.78–3.08 vs. high-SES without MetS; *p* for interaction < 0.001), with positive additive interaction (RERI 0.68). **Conclusions**: Over two decades, low SES amplified the CAD burden associated with MetS. Adults living in the most severe material deprivation who also had MetS constituted the highest risk group, of direct relevance to risk stratification and disparities in emergency and acute cardiovascular care.

## 1. Introduction

Cardiovascular disease remains the leading cause of death worldwide, and coronary artery disease (CAD) accounts for the largest share of this burden and of acute cardiovascular presentations to emergency departments [1]. Emergency and acute care physicians are frequently the first clinicians to encounter patients with undifferentiated chest pain or established CAD, making the early identification of high-risk populations a clinically meaningful goal that extends from the acute presentation back to primordial and primary prevention [1].

The public health weight of this problem is difficult to overstate. Ischemic heart disease is the single largest contributor to global years of life lost, and its incidence is rising in rapidly ageing East Asian societies, including the Republic of Korea, where demographic transition and the westernization of diet and activity patterns have produced a sustained increase in the metabolic precursors of atherosclerosis [1,2,3]. For the emergency and acute care physician, CAD is not an abstract epidemiological entity but a daily clinical reality: acute coronary syndromes are among the most time-critical presentations in the emergency department, chest pain is one of the commonest reasons for acute attendance, and the disposition and downstream trajectory of these patients depend heavily on their underlying risk profile. Understanding which populations carry the greatest long-term burden of CAD is therefore directly relevant to how acute care systems triage, investigate, and follow up the patients they encounter.

Metabolic syndrome (MetS)—the clustering of abdominal obesity, dysglycemia, dyslipidemia, and elevated blood pressure—approximately doubles the long-term risk of cardiovascular events and death [4,5,6]. Standardized diagnostic criteria have been harmonized across international bodies [7,8], and the prevalence of MetS has risen steadily in both Western and Asian populations, including the Republic of Korea [2,3]. As a modifiable, readily measurable phenotype, MetS is an attractive axis for cardiovascular risk stratification.

Independent of metabolic factors, socioeconomic status (SES) exhibits a robust inverse association with cardiovascular morbidity and mortality across diverse health systems [9,10,11,12,13,14]. Lower SES has been linked to adverse health behaviors, reduced access to preventive care, chronic psychosocial stress, and worse cardiovascular outcomes, and these gradients persist even where universal health coverage is in place [10,12,13]. In Korea, socioeconomic differences in mortality and major causes of death have been documented [15]. More recent national data confirm that these disparities have not been resolved; although age-standardized cardiovascular mortality in Republic of Korea declined substantially over the past three decades, the absolute burden of heart disease has risen again since the early 2000s [16]. A systematic review of Korean studies found persistent socioeconomic gradients across virtually every domain of cardiovascular health, including risk factor prevalence, treatment, and outcomes [17]. Importantly, SES is also associated with the prevalence and components of MetS itself, and these associations may differ by sex and population [18,19].

A plausible framework for why SES should not merely add to, but actively modify, metabolic risk is the concept of allostatic load. Chronic socioeconomic adversity—financial strain, job insecurity, and limited control over one’s environment—produces sustained activation of the hypothalamic–pituitary–adrenal axis and the sympathetic nervous system. The resulting long-term elevation in cortisol and catecholamines promotes visceral fat deposition, insulin resistance, and a low-grade pro-inflammatory state, the very substrates that define MetS [10,12]. Under this model, the same metabolic phenotype may carry different biological consequences depending on the socioeconomic environment in which it is sustained, providing a mechanistic rationale for testing interactions rather than simple additions.

Two features of this question require a long observation window and a particular health system context. First, atherosclerosis develops over decades, and the pathways through which socioeconomic disadvantage acts—cumulative stress exposure, sustained differences in risk factor control, and repeated interruptions in preventive care—accumulate rather than act at a single point in time. Short-to-intermediate follow-up may therefore capture only the earliest and most attenuated portion of the divergence, whereas a two-decade horizon allows cumulative risk to become manifest. Second, the Republic of Korea provides an informative setting because universal health insurance covers essentially the entire population, largely removing the insurance barrier that confounds socioeconomic comparisons in many other countries. That marked disparities nonetheless persist in Republic of Korea implies that non-financial mechanisms—health literacy, time and mobility constraints, out-of-pocket cost-sharing, differential quality and continuity of care, and psychosocial stress—remain operative, and that expanding nominal coverage alone is insufficient to close the gap [12,13,17].

Despite extensive evidence on MetS and on SES separately, whether SES modifies the long-term association between MetS and CAD remains insufficiently characterized, particularly over multi-decade horizons and in settings with universal insurance. Clarifying this interaction is relevant to emergency and acute care medicine, where recognizing socioeconomically vulnerable patients with metabolic risk could inform risk stratification, disposition, and linkage to longitudinal preventive care. Using a nationwide cohort of nearly half a million Korean adults followed for up to 20 years, we examined the independent and joint associations of MetS and income-based SES with incident CAD and tested for an interaction between them.

## 2. Materials and Methods

### 2.1. Data Source and Study Population

**Figure 1:**
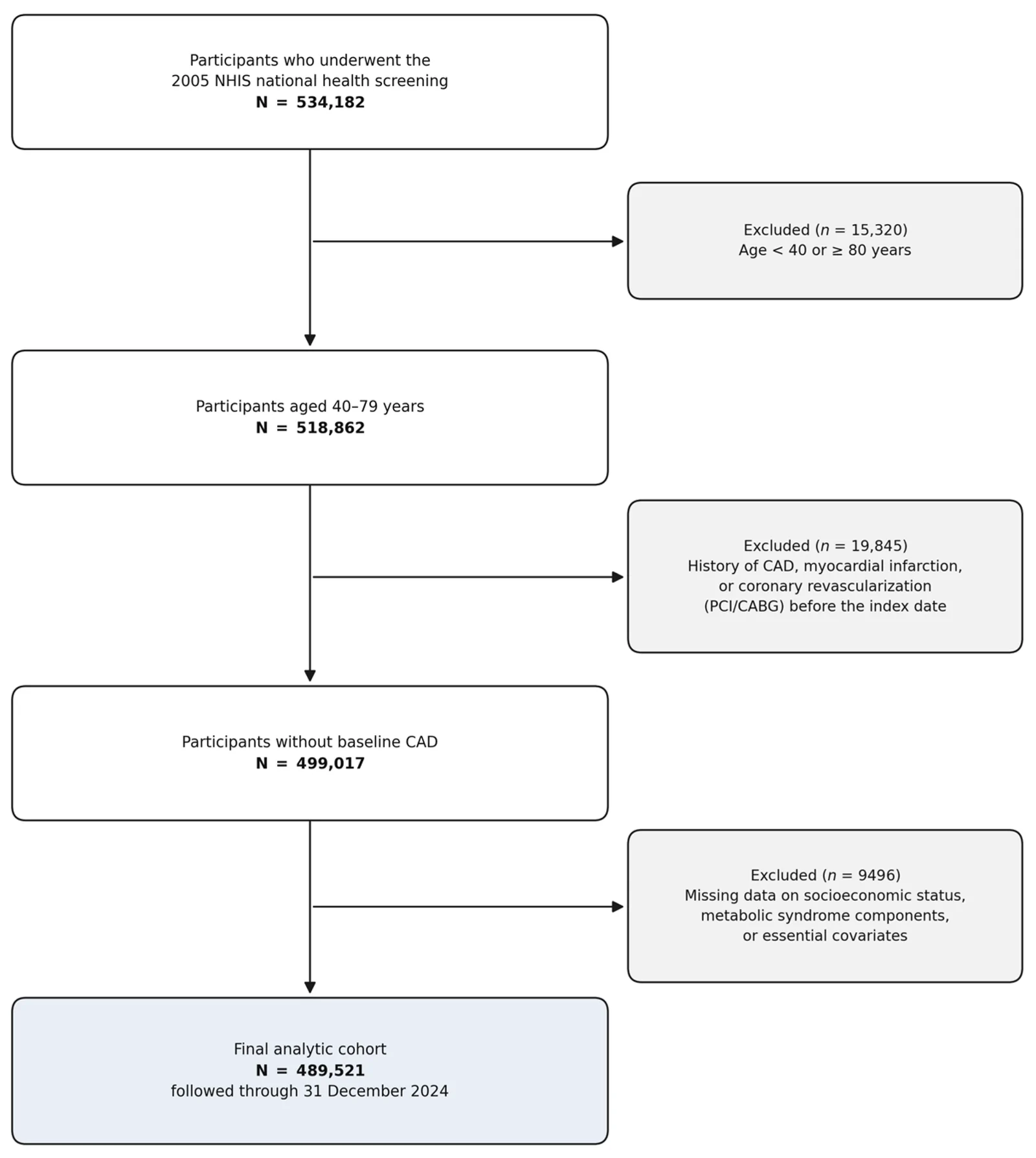
Flow diagram of participant selection. Of 534,182 adults who underwent the 2005 national health screening of the Korean National Health Insurance Service, 15,320 were excluded for age outside the 40–79-year range, 19,845 for a history of coronary artery disease, myocardial infarction, or coronary revascularization (percutaneous coronary intervention or coronary artery bypass grafting) before the index date, and 9496 for missing data on socioeconomic status, metabolic syndrome components, or essential covariates, leaving a final analytic cohort of 489,521 participants. CAD, coronary artery disease; CABG, coronary artery bypass grafting; NHIS, National Health Insurance Service; PCI, percutaneous coronary intervention.

This nationwide retrospective cohort study used the Korean National Health Insurance Service (NHIS) database, which covers virtually the entire population of the Republic of Korea and contains sociodemographic information, national health examination results, and healthcare-utilization records coded by the International Classification of Diseases, 10th Revision (ICD-10) [20,21]. The NHIS has been widely validated as a resource for population-based epidemiological research [20,21]. The retrospective study period spanned 1 January 2004 to 31 December 2024. The source population comprised adults who underwent a national general health examination in 2005, which served as the baseline. Participants were included if they were aged 40–79 years at baseline. We applied the following sequential exclusion criteria: (i) age outside the 40–79-year range; (ii) prevalent CAD, defined as any inpatient or outpatient claim carrying a diagnostic code for ischemic heart disease (ICD-10 I20–I25) or any claim for coronary revascularization—percutaneous coronary intervention or coronary artery bypass grafting—during a one-year washout period preceding baseline (1 January to 31 December 2004) or at the baseline examination itself; and (iii) missing data on any examination measurement, insurance premium, or covariate required for the analysis. Because prior coronary revascularization identifies established atherosclerotic disease with a high risk of recurrent events, procedure codes were used in addition to diagnostic codes so that such individuals were removed from the cohort at risk. The stepwise derivation of the analytic cohort is shown in Figure 1. After applying these criteria, 489,521 participants were followed from the baseline examination for up to 20 years (approximately 8.9 million person-years) through 31 December 2024. Institutional Review Board approval was obtained before data access and analysis.

### 2.2. Definition of Metabolic Syndrome

MetS was defined according to the harmonized criteria [7] as the presence of at least three of the following five components: (i) general obesity, used as a surrogate for abdominal obesity and defined as a BMI ≥ 25 kg/m^2^, the threshold for obesity in Korean adults recommended by the Korean Society for the Study of Obesity [22]; (ii) elevated blood pressure (≥130/85 mmHg or use of antihypertensive medication); (iii) elevated fasting glucose (≥100 mg/dL or use of glucose-lowering medication); (iv) elevated triglycerides (≥150 mg/dL); and (v) reduced high-density lipoprotein (HDL) cholesterol (<40 mg/dL in men, <50 mg/dL in women). Examination measurements and prescription/claims records were used to ascertain each component. We note explicitly that waist circumference was not recorded in the 2005 national health examination dataset; BMI was therefore substituted for waist circumference, and the resulting definition is a modification of, rather than a strict application of, the harmonized criteria. The implications of this substitution are addressed in the Limitations.

### 2.3. Assessment of Socioeconomic Status

**Figure 2:**
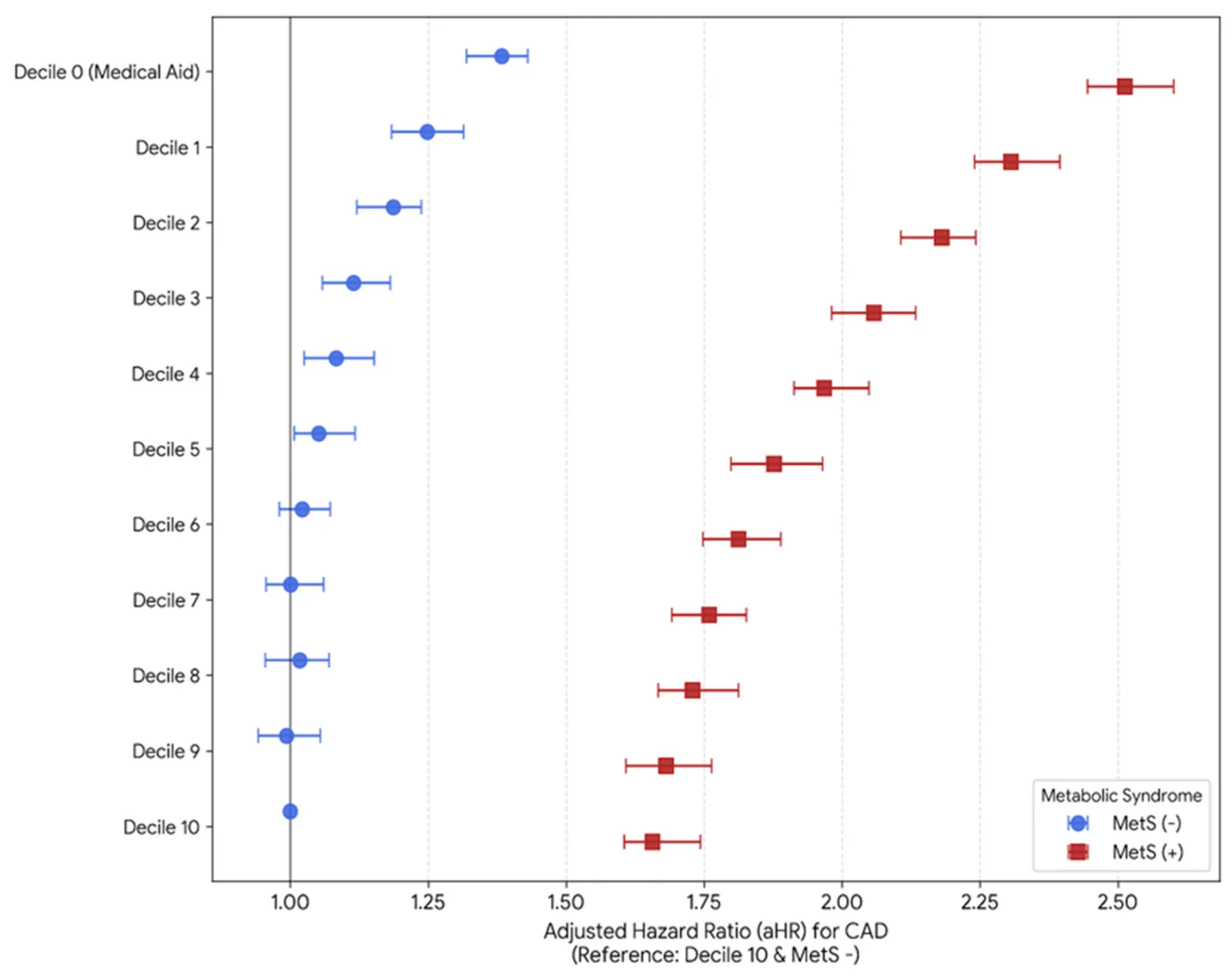
Comparison of CAD risk by metabolic syndrome status across socioeconomic deciles. Adjusted hazard ratios (aHRs) with 95% confidence intervals for incident coronary artery disease (CAD) are shown for participants without (MetS−) and with (MetS+) metabolic syndrome across income-based premium deciles, with Decile 10 and MetS (−) as the reference. CAD, coronary artery disease; MetS, metabolic syndrome.

SES was operationalized using the income-based health insurance premium, a commonly used proxy in NHIS research, expressed in deciles. Decile 0 denoted Medical Aid beneficiaries (the lowest income group, whose contributions are subsidized by the government), and Deciles 1–10 represented increasing premium-based income. For the principal analyses, SES was classified into four levels: high (deciles 8–10), middle (deciles 4–7), low income (deciles 1–3), and Medical Aid (decile 0). Because Medical Aid beneficiaries represent a distinct population living in extreme poverty, combining them with deciles 1–3—as is common in previous NHIS-based studies—could dilute their specific risk; they were therefore retained as a separate category throughout, and the full decile resolution is additionally shown in Figure 2.

### 2.4. Outcome Ascertainment

The primary outcome was incident CAD during follow-up, defined as a new diagnosis of myocardial infarction (ICD-10 codes I21–I23) or angina pectoris (I20). The first qualifying event after the baseline examination was used to define the time to event; participants without an event were censored at death, disenrollment, or the end of follow-up. To examine whether the associations were consistent across clinical presentations, myocardial infarction and angina pectoris were additionally analyzed as separate outcomes.

### 2.5. Covariates

Covariates comprised age, sex, lifestyle factors (current smoking, alcohol consumption ≥ 2 times/week, and regular exercise), body mass index, comorbidities (hypertension, diabetes mellitus, dyslipidemia, and chronic kidney disease), and examination/laboratory measurements obtained at baseline. Lifestyle factors were ascertained from the standardized self-administered questionnaire completed at the national health examination. Current smoking was defined as a self-report of smoking at the time of the examination; the questionnaire did not capture cumulative exposure, so pack-years were not available. Alcohol consumption was defined as drinking on two or more occasions per week; the questionnaire recorded drinking frequency only, and no information on the volume or grams of alcohol consumed per occasion was available. Regular exercise was defined as engaging in vigorous-intensity physical activity on two or more occasions per week. All three variables were therefore self-reported and frequency-based rather than quantitative.

### 2.6. Statistical Analysis

Baseline characteristics were compared across groups using the χ^2^ test for categorical variables and analysis of variance for continuous variables. Incidence rates were calculated as the number of events per 1000 person-years. Cox proportional hazards regression was used to estimate hazard ratios (HRs) and 95% confidence intervals (CIs) for CAD. Models were built in nested fashion, and the fully adjusted model—including age, sex, smoking, alcohol consumption, regular exercise, body mass index, chronic kidney disease, and the alternate exposure (SES in the metabolic syndrome analysis and metabolic syndrome status in the SES analysis)—is presented throughout. To evaluate joint effects, a six-level exposure variable combining SES group and MetS status was modeled with high-SES/non-MetS participants as the reference, using the Model 3 covariate set. Interactions were assessed on the multiplicative scale (product term) and on the additive scale by the relative excess risk due to interaction (RERI). Twenty-year cumulative incidence curves were constructed for the six joint-exposure groups. The proportional hazards assumption was examined graphically and with Schoenfeld residuals. Three additional analyses were prespecified for this revision. First, because sex differences in both MetS and cardiovascular risk are well established, all models were repeated separately in men and women, and a multiplicative interaction with sex was formally tested. Second, myocardial infarction and angina pectoris were modeled as separate outcomes to assess consistency across CAD phenotypes. Third, Medical Aid beneficiaries (decile 0) were analyzed separately from deciles 1–3 in a four-level SES variable to determine whether the gradient was driven by extreme poverty. All tests were two-sided, with *p* < 0.05 considered statistically significant. Because several exposure–outcome and interaction hypotheses were examined, the principal interaction tests were prespecified and limited in number, and the significance of the main findings was evaluated in light of the multiplicity of comparisons; secondary subgroup analyses are accordingly interpreted as exploratory rather than confirmatory. To guard against type I error inflation, a Bonferroni correction was applied across the four interaction tests reported (the overall metabolic syndrome × socioeconomic status interaction, the three-way interaction with sex, and the two sex-specific interactions), giving a corrected significance threshold of α = 0.0125. All analyses were performed using R version 4.4.1 (R Foundation for Statistical Computing, Vienna, Austria) with the survival, survminer, and ggplot2 packages.

### 2.7. Ethics Statement

The study was conducted in accordance with the Declaration of Helsinki and approved by the Institutional Review Board of Chonnam National University Hospital (approval code CNUH-EXP-2025-082; date of approval 28 March 2025). Because the analysis used anonymized, de-identified data, the requirement for informed consent was waived.

## 3. Results

### 3.1. Baseline Characteristics

Among 489,521 participants, 149,737 (30.6%) had MetS at baseline. Compared with participants without MetS, those with MetS were older (57.7 ± 7.4 vs. 52.4 ± 8.2 years), less likely to be male, and had a markedly higher burden of hypertension (81.4% vs. 28.9%), diabetes mellitus (49.1% vs. 6.6%), and dyslipidemia (70.5% vs. 32.7%), together with a higher body mass index, blood pressure, fasting glucose, and triglycerides and lower HDL cholesterol (all *p* < 0.001; Table 1).

When participants were classified by SES, the lowest SES group had the highest prevalence of MetS (38.4% in low vs. 24.9% in high SES), was older, smoked more, exercised less, and carried a greater comorbidity burden, while the highest SES group reported more frequent alcohol consumption (all *p* < 0.001; Table 2). The 20-year incidence of CAD increased stepwise as SES decreased, from 7.8% in the high-SES group to 12.4% in the low-SES group. When Medical Aid beneficiaries were considered separately, they accounted for 28,390 participants (5.8%) and showed the highest MetS prevalence of any stratum (13,160 of 28,390; 46.4%; Table 1).

### 3.2. Independent Associations of Metabolic Syndrome and Socioeconomic Status with CAD

During follow-up, 48,428 participants (9.9%) developed CAD, including 13,619 myocardial infarctions (2.8%) and 34,809 cases of angina pectoris (7.1%). The crude incidence rate of CAD was 8.92 per 1000 person-years among participants with MetS, compared with 3.89 per 1000 person-years among those without MetS—a more than twofold difference. In the fully adjusted model, MetS remained strongly associated with incident CAD (aHR 1.84; 95% CI 1.78–1.91; Table 3). A graded socioeconomic gradient was also evident; relative to participants in the highest premium deciles, the adjusted hazard increased for those in middle deciles (aHR 1.15; 95% CI 1.11–1.19), in the low-income deciles (1.28; 1.23–1.33), and among Medical Aid beneficiaries (1.62; 1.54–1.70), with corresponding incidence rates of 4.24, 5.37, 6.17, and 8.75 per 1000 person-years (Table 3).

### 3.3. Joint Association and Interaction

In the joint analysis using high-SES participants without MetS as the reference, risk increased across both exposures (Table 4, Figure 2). Among participants without MetS, the socioeconomic gradient was present but modest across the premium deciles (middle 1.12, 95% CI 1.08–1.16; low income 1.25, 1.20–1.31), rising more steeply only among Medical Aid beneficiaries (1.56; 1.46–1.67). Among participants with MetS, the gradient was consistently steeper, increasing from 1.68 (1.62–1.74) in the highest deciles to 1.90 (1.84–1.96) in middle deciles, 2.18 (2.11–2.26) in low-income deciles, and 2.92 (2.78–3.08) among Medical Aid beneficiaries, who constituted the highest risk group (*p* for interaction < 0.001). Incidence rates mirrored this pattern, increasing from 3.13 per 1000 person-years in the high-SES group without MetS to 11.40 per 1000 person-years among Medical Aid beneficiaries with MetS. The relative excess risk due to interaction was positive (RERI 0.68), indicating that the joint effect of severe socioeconomic disadvantage and MetS exceeded the sum of their individual effects. Notably, separating Medical Aid beneficiaries from the remaining low-income deciles revealed a substantially steeper gradient than a combined low-SES category would have shown, indicating that pooling these groups attenuates rather than exaggerates the socioeconomic gradient. The decile-resolution pattern in Figure 2 was concordant: within every premium decile, MetS conferred a higher hazard, and the hazard rose progressively from the highest decile to Decile 0 (Medical Aid).

### 3.4. Cumulative Incidence over 20 Years

**Figure 3:**
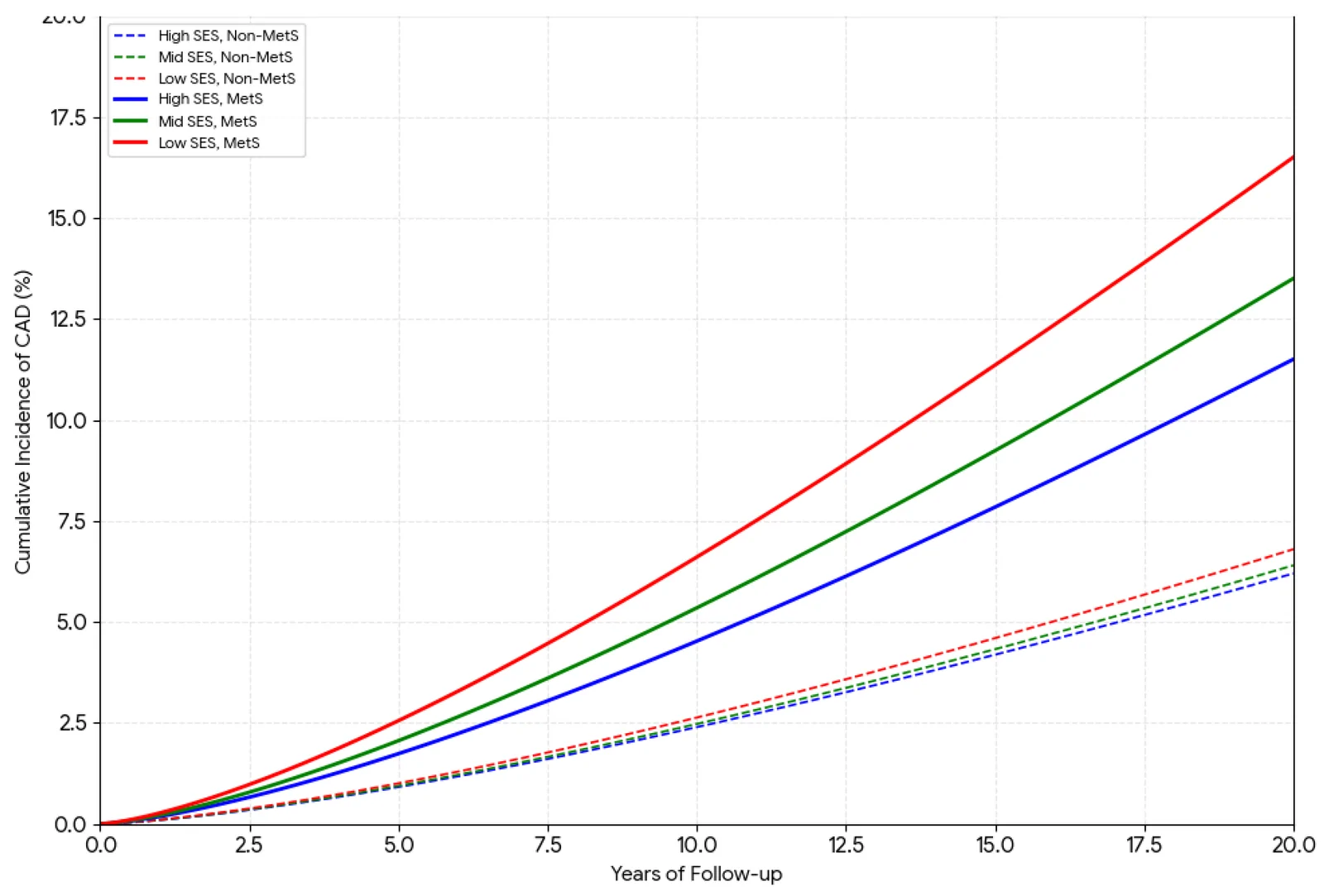
Twenty-year cumulative incidence curves for coronary artery disease. Cumulative incidence of coronary artery disease (CAD) over 20 years of follow-up is shown for six groups defined by socioeconomic status (high, middle, low) and metabolic syndrome status (dashed lines, non-MetS; solid lines, MetS). CAD, coronary artery disease; MetS, metabolic syndrome; SES, socioeconomic status.

The 20-year cumulative incidence curves diverged early and widened progressively over follow-up (Figure 3). Curves for participants with MetS lay consistently above those without MetS within every SES stratum, and within each MetS stratum the low-SES curve was highest. By 20 years, the estimated cumulative incidence of CAD reached approximately 16.5% in the low-SES/MetS group, compared with roughly 6% in the non-MetS groups, illustrating the compounding of socioeconomic and metabolic risk over the long term.

### 3.5. Sex-Stratified Analyses

Analyses repeated separately in men (n = 252,818) and women (n = 236,703) showed the same qualitative pattern in both sexes but a markedly steeper socioeconomic gradient in women (Table 5). Among participants with MetS, the adjusted hazard rose from 1.62 (95% CI 1.53–1.71) in high-SES men to 1.95 (1.85–2.06) in low-income men and 2.28 (2.12–2.45) in men receiving Medical Aid. In women, the corresponding values rose from 1.74 (1.62–1.86) to 2.58 (2.42–2.75) and 3.42 (3.20–3.65). A parallel but weaker gradient was seen among participants without MetS (Medical Aid aHR 1.45 in men and 1.75 in women). The multiplicative interaction between MetS and SES was nominally significant in men (*p* = 0.038) and strongly significant in women (*p* < 0.001), and the three-way interaction between MetS, SES, and sex was also significant (*p* = 0.012). After Bonferroni correction for the four interaction tests (α = 0.0125), the overall interaction (*p* < 0.001), the three-way interaction with sex (*p* = 0.012; corrected *p* = 0.048), and the interaction among women (*p* < 0.001) remained statistically significant, whereas the weaker interaction among men (*p* = 0.038; corrected *p* = 0.152) did not—consistent with a modifying effect concentrated among women. All principal hazard ratios were associated with *p* < 0.0001 and remained significant under the same correction.

### 3.6. Consistency Across CAD Phenotypes

When myocardial infarction and angina pectoris were analyzed as separate outcomes, the joint pattern was reproduced for both phenotypes (Table 6). Relative to high-SES participants without MetS, Medical Aid beneficiaries with MetS had an adjusted hazard of 3.05 (95% CI 2.78–3.34) for myocardial infarction and 2.48 (2.33–2.64) for angina pectoris; the corresponding values in the low-income stratum were 2.34 (2.16–2.53) and 2.05 (1.95–2.15). Within every stratum, the gradient was somewhat steeper for myocardial infarction than for angina pectoris, but the direction and ordering of risk were identical, indicating that the modifying effect of socioeconomic status was not confined to a single clinical presentation of CAD and is unlikely to be an artifact of differential coding of the softer endpoint.

## 4. Discussion

In this nationwide cohort of 489,521 Korean adults followed for up to two decades, MetS and low SES were each independently associated with incident CAD, and their combination produced the greatest risk. Medical Aid beneficiaries with MetS had a nearly threefold higher adjusted hazard of CAD than high-SES adults without MetS (2.92; 95% CI 2.78–3.08), and the positive additive interaction (RERI 0.68) indicated that the socioeconomic disadvantage amplified the metabolic contribution to CAD beyond what would be expected from the two exposures acting independently.

The magnitude of the MetS–CAD association is consistent with prior meta-analyses reporting an approximate doubling of cardiovascular risk with MetS [4,5,6]. Likewise, the inverse SES gradient aligns with a large body of evidence linking lower socioeconomic position to higher cardiovascular morbidity and mortality across health systems [9,10,11,12,13,14]. Our findings extend this literature in three respects: the very long (20-year) horizon, the setting of universal health insurance, and the explicit evaluation of interaction between metabolic and socioeconomic risk, which together suggest that low SES is not merely an additive risk marker but a modifier of metabolic risk for CAD. Two further observations support this interpretation. First, the pattern held for both myocardial infarction and angina pectoris when these were modeled separately, with a somewhat steeper gradient for myocardial infarction (3.05 versus 2.48 in the most disadvantaged stratum with MetS), indicating that the finding is not an artifact of how the composite endpoint was assembled or of differential coding of the softer diagnosis. Second, analyzing Medical Aid beneficiaries separately from the remaining low-income deciles showed that risk was concentrated in those living in the most extreme material deprivation (aHR 2.92; 95% CI 2.78–3.08 among those with MetS), so the conventional practice of pooling these groups attenuates rather than manufactures the socioeconomic gradient.

Several mechanisms may underlie the steeper metabolic gradient observed among socioeconomically disadvantaged participants. Lower SES was accompanied by a higher prevalence of smoking, physical inactivity, obesity, and comorbid conditions, consistent with differential exposure to behavioral and clinical risk factors that account for the majority of the population burden of myocardial infarction worldwide [13,18,19,23]. Beyond measured behaviors, socioeconomic disadvantage is associated with chronic psychosocial stress and allostatic load, delayed presentation, and less intensive risk factor control, all of which could accelerate the progression from metabolic dysfunction to clinical CAD [10,12]. The persistence of these gradients despite universal insurance coverage indicates that financial access alone is insufficient to eliminate disparities and that non-financial barriers remain important [12,13].

The physiology linking chronic disadvantage to accelerated atherogenesis is increasingly well characterized. Prolonged psychosocial stress produces sustained activation of the hypothalamic–pituitary–adrenal (HPA) axis, with loss of the normal diurnal cortisol rhythm and persistent hypercortisolemia. Excess glucocorticoid exposure antagonizes insulin signaling in skeletal muscle and liver, promotes lipolysis and the redistribution of adipose tissue to the visceral compartment, and thereby directly worsens insulin resistance and atherogenic dyslipidemia—precisely the components that constitute MetS. In parallel, chronic stress and expanded visceral adipose tissue drive the release of pro-inflammatory cytokines such as interleukin-6 and tumor necrosis factor-α, which impair endothelial nitric oxide bioavailability, increase endothelial adhesion molecule expression, and facilitate monocyte recruitment into the arterial wall. Sympathetic overactivity adds further hemodynamic shear stress and pro-thrombotic activation. The net effect is that, for an equivalent metabolic phenotype, individuals living under sustained socioeconomic adversity plausibly experience faster plaque initiation and progression and greater plaque instability. This mechanistic sequence offers a coherent biological explanation for the positive additive interaction observed here, in which MetS conferred a substantially steeper hazard among low-SES than among high-SES participants.

Cardiovascular risk is determined not only by baseline risk factor burden but also by the extent to which guideline-directed preventive therapies are actually implemented and sustained. Contemporary preventive strategies—high-intensity statins and non-statin lipid-lowering agents, antithrombotic therapy, renin–angiotensin system blockade, and, more recently, sodium–glucose cotransporter-2 inhibitors and glucagon-like peptide-1 receptor agonists—substantially reduce, but do not abolish, the residual risk of ischemic events, and their benefit depends critically on access, adherence, and continuity of care [24]. Socioeconomically disadvantaged individuals are systematically less likely to receive, intensify, and remain on such therapies, so part of the socioeconomic gradient observed in this cohort may reflect differential implementation of preventive care rather than differences in intrinsic biological risk alone. Because pharmacy claims for preventive medications were not incorporated into our models, we could not quantify this contribution, and the estimates reported here should be interpreted as the net effect of biological and treatment-related pathways combined. This interpretation reinforces rather than weakens the clinical message: the low-SES/MetS group is precisely the population in which systematic optimization of secondary and primordial prevention is most likely to yield benefit. In particular, differential use of renin–angiotensin–aldosterone system inhibitors and statins, and differences in the adequacy of their dosing and the persistence of treatment, are plausible contributors to the socioeconomic gradient observed here; unfortunately, the corresponding prescription and dose-adequacy data were not available in our extract, and this remains an important direction for future work.

Sex is a further potential modifier of these relationships. Socioeconomic disparities in MetS have been reported to differ between men and women, with stronger inverse gradients described in women in several populations [18,19], and Korean cardiovascular studies have likewise documented sex-specific patterns of risk factor burden and outcome [16,17]. We therefore repeated all analyses separately in men and women (Table 5). The direction of the association was identical in both sexes, but its magnitude was not. Among men with MetS, the adjusted hazard rose from 1.62 (95% CI 1.53–1.71) in the highest premium deciles to 2.28 (2.12–2.45) among Medical Aid recipients. Among women with MetS, the same gradient was appreciably steeper, rising from 1.74 (1.62–1.86) to 3.42 (3.20–3.65). The interaction between MetS and SES was nominally significant in men (*p* = 0.038) and strongly significant in women (*p* < 0.001); after Bonferroni correction for the multiple interaction tests, the interaction in men was no longer significant, whereas that in women, the overall interaction, and the three-way MetS × SES × sex interaction (*p* = 0.012) remained significant, indicating that the modifying effect of socioeconomic disadvantage on metabolic risk was not homogeneous across sexes. Women receiving Medical Aid who also had MetS were the single highest risk subgroup identified in this cohort. These findings are consistent with NHANES data showing that socioeconomic gradients in MetS are more pronounced among women [18,19]. Several explanations are plausible: women in this birth cohort were more likely to be economically dependent, so Medical Aid status may capture more profound material deprivation in women than in men; competing demands of caregiving may delay symptom evaluation; and the loss of estrogen-mediated vascular protection after menopause may interact with metabolic and psychosocial adversity. An important methodological caveat is that, in the Korean system, many women are enrolled as dependents, so their premium decile reflects household rather than personal income; this may render the SES measure less precise—but not less real—in women. Taken together, these results suggest that socioeconomically disadvantaged women with MetS warrant particular attention in cardiovascular risk stratification.

These observations carry practical implications for emergency and acute care medicine, the focus of this special issue. Emergency departments are a critical—and sometimes the only—point of contact with the health system for socioeconomically vulnerable patients, including those who present with chest pain or acute coronary syndromes. Recognizing that low-SES patients with metabolic risk constitute a particularly high-risk group could inform risk communication, threshold for investigation, and, importantly, structured linkage from the acute encounter to longitudinal preventive care and risk factor management. Embedding socioeconomic context into cardiovascular risk stratification may help acute care systems contribute to narrowing, rather than perpetuating, cardiovascular health disparities [1,12].

Several concrete applications follow from these findings. First, the socioeconomic and metabolic profiles identified here are composed entirely of variables that are already available, or readily obtainable, at the point of acute care: a body-mass-index-based metabolic phenotype and an administrative marker of income or insurance status. This raises the practical possibility of flagging the highest risk group—socioeconomically disadvantaged patients with metabolic syndrome, and disadvantaged women in particular—at the time of an index emergency-department contact, so that the acute encounter can serve as an entry point to structured secondary and primordial prevention rather than an isolated episode of care. Second, our results argue for incorporating a measure of socioeconomic context into cardiovascular risk-stratification tools, which conventionally rely on biological risk factors alone and may therefore systematically underestimate risk in the most disadvantaged patients. Third, the concentration of risk among Medical Aid beneficiaries provides a quantitative rationale for targeted, resource-efficient interventions—medication-adherence support, subsidized cardioprotective pharmacotherapy, and proactive follow-up—directed at a defined and readily identifiable population rather than at the population at large. Because these levers operate at the interface of acute and longitudinal care, emergency and acute care services are well positioned to contribute to closing, rather than perpetuating, the cardiovascular disparities documented here.

The strengths of this study include its nationwide scope, very large sample size, prolonged follow-up, and the use of an objective, income-based measure of SES derived from insurance premiums in a universal coverage system, which mitigates several biases inherent to self-reported socioeconomic measures [18,19]. The analysis of both multiplicative and additive interactions provides a more complete picture of how the two exposures combine.

Our findings also delineate several priorities for future research. The most immediate is to link longitudinal prescription and adherence data—statins, renin–angiotensin–aldosterone system inhibitors, antithrombotic agents, and newer cardiometabolic therapies—to quantify how much of the socioeconomic gradient reflects differential implementation of guideline-directed prevention rather than intrinsic biological risk, a question our data could raise but not resolve. A second priority is to model socioeconomic status and metabolic status as time-varying exposures, so that income mobility, retirement, and change in metabolic phenotype over a multi-decade horizon can be captured rather than fixed at baseline. A third is to extend the analysis to waist circumference-based and biomarker-based definitions of metabolic syndrome as these measurements become available in more recent screening cohorts, and to examine cause-specific and competing-risk outcomes. Finally, the pronounced sex difference we observed—together with the dependency-based structure of insurance premiums in women—warrants dedicated study of how best to measure and act on socioeconomic risk in women, and prospective evaluation of whether interventions targeted at the highest risk socioeconomic–metabolic subgroups can measurably narrow the disparity in CAD incidence.

This study also has limitations. First, outcomes and comorbidities were ascertained from administrative claims using ICD-10 codes, which may be subject to misclassification. Although prevalent CAD was excluded using both diagnostic codes and coronary revascularization procedure codes, the washout period preceding baseline was one year; individuals with established but clinically quiescent coronary disease who happened not to use medical services during 2004 could therefore have been misclassified as incident cases. Such residual misclassification would be expected to be non-differential with respect to MetS status and would most plausibly bias the estimates toward the null, although differential misclassification cannot be formally excluded if low-SES individuals used health services less frequently during the washout window. Second, waist circumference was not available in the 2005 examination dataset, so BMI ≥ 25 kg/m^2^ was substituted as the obesity criterion. This is a recognized modification of the harmonized definition; because BMI does not distinguish visceral from subcutaneous or lean mass, some participants with true central adiposity will have been misclassified, and the MetS phenotype analyzed here is best understood as a BMI-based approximation. Relatedly, BMI enters both the exposure definition and the covariate set in Models 2 and 3, which constitutes partial over-adjustment and may have attenuated the apparent MetS–CAD association; the fully adjusted estimate should therefore be interpreted as conservative. Third, we could not determine whether the association of MetS with CAD is independent of, or merely a summary of, its individual components, because analyses evaluating the incremental prognostic value of the syndrome beyond its constituent risk factors were not feasible in this dataset; the reported estimates should therefore be read as the effect of the composite phenotype rather than as evidence that the syndrome carries information beyond its parts. Fourth, lifestyle covariates were self-reported and frequency-based, without pack-year, intensity, or alcohol-volume quantification, so residual confounding by these behaviors is likely. Fifth, both MetS and SES were assessed only at baseline. Over a 20-year horizon, individual socioeconomic position may change substantially through job loss, retirement, or income mobility, and metabolic status may improve or deteriorate; a time-varying covariate approach was considered but could not be implemented with the available data extract, so the estimates reflect baseline status and are subject to regression-dilution bias, again most plausibly toward the null. Premium-based SES is a proxy that does not fully capture education, wealth, or neighborhood context, and residual and unmeasured confounding cannot be excluded. The additive interaction was summarized by a point estimate (RERI) without a confidence interval, and the findings are associational; causal interpretation requires caution. Finally, the cohort is drawn from a single country with universal insurance, which may limit generalizability to other health systems, although it also strengthens internal validity by reducing access-related confounding. Finally, multiple exposure, outcome, and interaction analyses were performed; although the principal associations were highly significant (*p* < 0.001) and would withstand conventional corrections for multiple comparisons, we did not apply a formal family-wise adjustment, and the subgroup and phenotype analyses should therefore be regarded as hypothesis-generating.

## 5. Conclusions

In a nationwide Korean cohort followed for up to 20 years, MetS and low SES were independently associated with incident CAD, and low SES amplified the CAD burden associated with MetS, with the doubly exposed group at highest risk. Low-SES adults with MetS represent a readily identifiable high-risk population of direct relevance to risk stratification and to disparities in emergency and acute cardiovascular care. Integrating socioeconomic context into cardiovascular risk assessment and strengthening linkage from acute encounters to longitudinal prevention may help reduce these disparities. Taken together with the sex-specific and phenotype-specific analyses, these results identify a readily definable population—socioeconomically disadvantaged adults with metabolic syndrome, and disadvantaged women above all—in whom metabolic and social risk converge to produce the greatest long-term burden of CAD. Situating this population within the workflow of emergency and acute care medicine, and pairing recognition with targeted preventive action, offers a concrete opportunity to translate a persistent health inequality into an actionable clinical target.

## Figures and Tables

**Table 1:** Baseline characteristics of the study population according to metabolic syndrome status. Values are presented as mean ± standard deviation or *n* (%).

Characteristic	Total (N = 489,521)	Non-MetS (*n* = 339,784)	MetS (*n* = 149,737)	*p*-Value
Age, years	54.1 ± 9.2	52.4 ± 8.2	57.7 ± 7.4	<0.001
Male sex	252,818 (51.6)	182,937 (53.8)	69,881 (46.7)	<0.001
***Socioeconomic status***				
252,818 (51.6) 182,937 (53.8) 69,881 (46.7) <0.001				
High SES (deciles 8–10)	139,024 (28.4)	104,408 (30.7)	34,616 (23.1)	<0.001
Middle SES (deciles 4–7)	206,578 (42.2)	142,539 (41.9)	64,039 (42.8)	
Low income (deciles 1–3)	115,529 (23.6)	77,607 (22.8)	37,922 (25.3)	
Medical Aid (decile 0)	28,390 (5.8)	15,230 (4.5)	13,160 (8.8)	
***Lifestyle factors***				
Current smoker	159,602 (32.6)	119,156 (35.1)	40,446 (27.0)	<0.001
Alcohol (≥2/week)	198,581 (40.6)	149,316 (43.9)	49,265 (32.9)	<0.001
Regular exercise	130,455 (26.6)	101,040 (29.7)	29,415 (19.6)	<0.001
***Comorbidities***				
Hypertension	220,124 (45.0)	98,305 (28.9)	121,819 (81.4)	<0.001
Diabetes mellitus	96,116 (19.6)	22,528 (6.6)	73,588 (49.1)	<0.001
Dyslipidemia	216,775 (44.3)	111,233 (32.7)	105,542 (70.5)	<0.001
Chronic kidney disease	36,362 (7.4)	20,105 (5.9)	16,257 (10.9)	<0.001
***Examination and laboratory findings***				
Body mass index, kg/m^2^	24.12 ± 3.15	22.86 ± 2.48	27.05 ± 3.31	<0.001
Systolic BP, mmHg	128.9 ± 14.2	123.4 ± 13.6	141.2 ± 15.4	<0.001
Diastolic BP, mmHg	81.4 ± 9.4	78.2 ± 9.1	88.7 ± 10.3	<0.001
Fasting glucose, mg/dL	105.3 ± 31.2	95.4 ± 16.5	127.8 ± 41.2	<0.001
Total cholesterol, mg/dL	192.8 ± 35.7	188.5 ± 34.3	202.4 ± 38.8	<0.001
Triglycerides, mg/dL	142.9 ± 82.1	112.8 ± 58.6	211.2 ± 104.5	<0.001
HDL cholesterol, mg/dL	51.0 ± 10.8	54.9 ± 11.4	42.2 ± 9.7	<0.001
***20-year clinical outcomes***				
Coronary artery disease	48,428 (9.9)	24,173 (7.1)	24,255 (16.2)	<0.001
Myocardial infarction	13,619 (2.8)	6700 (2.0)	6919 (4.6)	<0.001
Angina pectoris	34,809 (7.1)	17,473 (5.1)	17,336 (11.6)	<0.001

MetS, metabolic syndrome; BP, blood pressure; HDL, high-density lipoprotein. *p*-values were derived from the χ^2^ test (categorical variables) and analysis of variance (continuous variables).

**Table 2:** Baseline characteristics of the study population according to socioeconomic status group. Values are presented as mean ± standard deviation or n (%).

Characteristic	Low SES (Decile 0–3; *n* = 143,919)	Middle SES (Decile 4–7; *n* = 206,578)	High SES (Decile 8–10; *n* = 139,024)	*p*-Value
Metabolic syndrome	55,299 (38.4)	63,964 (31.0)	34,585 (24.9)	<0.001
Age, years	56.2 ± 8.4	53.8 ± 8.8	52.1 ± 9.1	<0.001
Male sex	70,787 (49.2)	110,291 (53.4)	75,410 (54.2)	<0.001
***Lifestyle factors***				
Current smoker	55,551 (38.6)	63,478 (30.7)	37,286 (26.8)	<0.001
Alcohol (≥2/week)	48,448 (33.7)	88,378 (42.8)	63,402 (45.6)	<0.001
Regular exercise	27,744 (19.3)	56,548 (27.4)	47,791 (34.4)	<0.001
***Comorbidities***				
Hypertension	76,486 (53.1)	90,114 (43.6)	51,003 (36.7)	<0.001
Diabetes mellitus	33,873 (23.5)	39,750 (19.2)	19,887 (14.3)	<0.001
Dyslipidemia	68,487 (47.6)	90,819 (44.0)	54,092 (38.9)	<0.001
***Examination findings***				
Body mass index, kg/m^2^	24.82 ± 3.42	24.15 ± 3.12	23.64 ± 2.85	<0.001
Systolic BP, mmHg	132.4 ± 15.2	128.6 ± 14.3	124.7 ± 13.5	<0.001
Fasting glucose, mg/dL	110.4 ± 32.5	104.2 ± 28.4	98.7 ± 21.6	<0.001
***20-year clinical outcomes***				
Coronary artery disease	17,825 (12.4)	20,065 (9.7)	10,778 (7.8)	<0.001
Myocardial infarction	5423 (3.8)	5543 (2.7)	2587 (1.9)	<0.001
Angina pectoris	12,402 (8.6)	14,522 (7.0)	8191 (5.9)	<0.001

SES, socioeconomic status; BP, blood pressure. SES deciles were derived from income-based health insurance premiums (Decile 0 = Medical Aid). *p*-values were derived from the χ^2^ test (categorical variables) and analysis of variance (continuous variables).

**Table 3:** Association of metabolic syndrome and socioeconomic status with incident coronary artery disease.

Exposure	No. at Risk	Events	Person-Years	IR ^a^	aHR (95% CI) ^b^
Metabolic syndrome					
No	339,784	24,173	6,210,000	3.89	1.00 (Ref)
Yes	149,737	24,255	2,720,000	8.92	1.84 (1.78–1.91)
Socioeconomic status					
High SES (deciles 8–10)	139,024	10,850	2,560,000	4.24	1.00 (Ref)
Middle SES (deciles 4–7)	206,578	20,250	3,770,000	5.37	1.15 (1.11–1.19)
Low income (deciles 1–3)	115,529	12,955	2,100,000	6.17	1.28 (1.23–1.33)
Medical Aid (decile 0)	28,390	4373	500,000	8.75	1.62 (1.54–1.70)

aHR, adjusted hazard ratio; CI, confidence interval; IR, incidence rate; Ref, reference; SES, socioeconomic status. ^a^ IR is expressed per 1000 person-years. ^b^ Fully adjusted model: adjusted for age, sex, smoking, alcohol consumption, regular exercise, body mass index, chronic kidney disease, and the alternate exposure (socioeconomic status for the metabolic syndrome analysis; metabolic syndrome status for the socioeconomic status analysis). Socioeconomic status was classified into four levels, with Medical Aid beneficiaries analyzed separately from premium deciles 1–3.

**Table 4:** Joint association of socioeconomic status and metabolic syndrome with incident coronary artery disease.

Joint Exposure Group	No. at Risk	Events	Person-Years	IR ^a^	aHR (95% CI) ^b^
Without metabolic syndrome (n = 339,784)					
High SES (reference)	104,408	6035	1,930,000	3.13	1.00 (Ref)
Middle SES	142,539	10,215	2,620,000	3.90	1.12 (1.08–1.16)
Low income	77,607	6400	1,410,000	4.54	1.25 (1.20–1.31)
Medical Aid	15,230	1523	250,000	6.09	1.56 (1.46–1.67)
With metabolic syndrome (n = 149,737)					
High SES	34,616	4815	630,000	7.64	1.68 (1.62–1.74)
Middle SES	64,039	10,035	1,150,000	8.73	1.90 (1.84–1.96)
Low income	37,922	6555	690,000	9.50	2.18 (2.11–2.26)
Medical Aid	13,160	2850	250,000	11.40	2.92 (2.78–3.08)
p for interaction (MetS × SES)					<0.001

aHR, adjusted hazard ratio; CI, confidence interval; IR, incidence rate; SES, socioeconomic status. ^a^ IR is expressed per 1000 person-years. ^b^ aHRs were adjusted for age, sex, smoking, alcohol consumption, regular exercise, body mass index, and chronic kidney disease, with high-SES participants without metabolic syndrome as the reference. The relative excess risk due to interaction (RERI), calculated for the contrast of Medical Aid status and metabolic syndrome, was 0.68.

**Table 5:** Sex-stratified joint association of metabolic syndrome and socioeconomic status with incident coronary artery disease.

Joint Exposure Group	No. at Risk	Events	aHR (95% CI) ^a^
Men (n = 252,818)			
Without metabolic syndrome			
High SES (reference)	55,124	3980	1.00 (Ref)
Middle SES	74,300	6854	1.13 (1.08–1.18)
Low income	40,000	4200	1.25 (1.19–1.31)
Medical Aid	5215	723	1.45 (1.35–1.55)
With metabolic syndrome			
High SES	20,500	3615	1.62 (1.53–1.71)
Middle SES	33,520	7235	1.78 (1.69–1.87)
Low income	18,159	4200	1.95 (1.85–2.06)
Medical Aid	6000	1347	2.28 (2.12–2.45)
p for interaction (MetS × SES)			0.038
Women (n = 236,703)			
Without metabolic syndrome			
High SES (reference)	49,284	2055	1.00 (Ref)
Middle SES	68,239	3361	1.15 (1.08–1.23)
Low income	37,607	2200	1.34 (1.25–1.44)
Medical Aid	10,015	800	1.75 (1.62–1.89)
With metabolic syndrome			
High SES	14,116	1200	1.74 (1.62–1.86)
Middle SES	30,519	2800	2.15 (2.01–2.29)
Low income	19,763	2355	2.58 (2.42–2.75)
Medical Aid	7160	1503	3.42 (3.20–3.65)
p for interaction (MetS × SES)			<0.001
p for three-way interaction (MetS × SES × sex)			0.012

aHR, adjusted hazard ratio; CI, confidence interval; MetS, metabolic syndrome; Ref, reference; SES, socioeconomic status. ^a^ aHRs were adjusted for age, smoking, alcohol consumption, regular exercise, body mass index, and chronic kidney disease, with high-SES participants without metabolic syndrome of the same sex as the reference. Socioeconomic status was classified as high (deciles 8–10), middle (deciles 4–7), low income (deciles 1–3), and Medical Aid (decile 0). A Bonferroni-corrected significance threshold of α = 0.0125 was applied across the four interaction tests; the interaction among men (*p* = 0.038) did not remain significant after this correction.

**Table 6:** Joint association of metabolic syndrome and socioeconomic status with myocardial infarction and angina pectoris analyzed separately.

Joint Exposure Group	Total N	MI Events	MI aHR (95% CI) ^a^	Angina Events	Angina aHR (95% CI) ^a^
Without metabolic syndrome					
High SES (reference)	104,408	1600	1.00 (Ref)	4435	1.00 (Ref)
Middle SES	142,539	2750	1.18 (1.09–1.28)	7465	1.11 (1.05–1.17)
Low income	77,607	1800	1.38 (1.26–1.51)	4600	1.21 (1.15–1.28)
Medical Aid	15,230	550	1.72 (1.53–1.93)	973	1.51 (1.40–1.63)
With metabolic syndrome					
High SES	34,616	1450	1.62 (1.49–1.76)	3365	1.58 (1.49–1.68)
Middle SES	64,039	2900	1.95 (1.81–2.10)	7135	1.76 (1.68–1.85)
Low income	37,922	1850	2.34 (2.16–2.53)	4705	2.05 (1.95–2.15)
Medical Aid	13,160	719	3.05 (2.78–3.34)	2131	2.48 (2.33–2.64)

aHR, adjusted hazard ratio; CI, confidence interval; MI, myocardial infarction; Ref, reference; SES, socioeconomic status. ^a^ aHRs were adjusted for age, sex, smoking, alcohol consumption, regular exercise, body mass index, and chronic kidney disease, with high-SES participants without metabolic syndrome as the reference. Myocardial infarction was defined by ICD-10 codes I21–I23 and angina pectoris by I20; together they comprise the composite CAD endpoint (13,619 and 34,809 events, respectively).

## Data Availability

The data that support the findings of this study are available from the National Health Insurance Service (NHIS) of the Republic of Korea. Restrictions apply to the availability of these data, which were used under approval for the present study and are therefore not publicly available. Data may be requested from the NHIS (https://nhiss.nhis.or.kr) subject to its review and approval procedures.

## Abbreviations

CAD, coronary artery disease; CI, confidence interval; aHR, adjusted hazard ratio; HDL, high-density lipoprotein; ICD-10, International Classification of Diseases, 10th Revision; IR, incidence rate; MetS, metabolic syndrome; NHIS, National Health Insurance Service; RERI, relative excess risk due to interaction; SES, socioeconomic status.
